# Knowledge, Adherence, and Barriers to Gluten-Free Diet Among Adults with Celiac Disease in Saudi Arabia: A Pilot Study at a Tertiary Hospital in Madinah, Saudi Arabia

**DOI:** 10.3390/healthcare13101208

**Published:** 2025-05-21

**Authors:** May A. Alsayb, Suliman A. Alharbi, Renad H. Alloqmani, Ghaida A. Madkli, Rahaf A. Basharahil, Marah I. Alhejaili, Walaa A. Mumena

**Affiliations:** 1 Clinical Laboratory Sciences Department, College of Applied Medical Sciences, Taibah University, P.O. Box 344, Madinah 42353, Saudi Arabia; 2 Serology, Immunology & Tissue Typing Division, Laboratory & Blood Bank, King Fahad Hospital, Madinah 42351, Saudi Arabia; 3 Clinical Nutrition Department, College of Applied Medical Sciences, Taibah University, P.O. Box 344, Madinah 42353, Saudi Arabia

**Keywords:** celiac disease, adults, gluten-free diet, knowledge, adherence, barriers

## Abstract

**Background**: Celiac disease (CD) is an autoimmune condition triggered by gluten ingestion in genetically predisposed individuals. Management typically involves a strict gluten-free diet (GFD). However, there are limited data concerning adherence to GFD among adult CD patients in Saudi Arabia. Therefore, this study aimed to pilot test the assessment of knowledge, adherence to GFD, and barriers to adherence to GFD among adult celiac patients in Madinah, Saudi Arabia. **Methods**: Cross-sectional data were collected from 36 adults with celiac disease at King Fahad Hospital, Madinah (2021–2022). After obtaining consent, participants completed an online questionnaire covering sociodemographic data, GFD knowledge, adherence, and related barriers. **Results**: Only 33% of participants were aware of the Ministry of Health’s GFD support program, with 30.6% utilizing gluten-free products and 27.8% receiving financial assistance. Higher adherence scores were significantly associated with awareness of the program, reading nutrition labels, understanding GFD requirements, receiving financial support, and using separate utensils for gluten-free food preparation. The majority (58.3%) had not consulted a dietitian, and no significant association was found between dietitian consultation and GFD adherence. Poor knowledge and difficulty interpreting nutrition labels were reported as primary barriers. **Conclusions**: Improving public and patient awareness of the GFD and available support programs is essential in enhancing adherence among CD patients in Saudi Arabia. Healthcare providers should play a more active role in patient education and ongoing support.

## 1. Introduction

Celiac disease (CD), a chronic autoimmune enteropathy, manifests in genetically predisposed individuals through an aberrant immunological reaction to gluten [1]. The global prevalence of CD is 1.4%, and the prevalence is estimated to be higher in females than in males and significantly higher in children than in adults [2]. Data suggest that Saudi Arabia is one of the countries with a high prevalence of CD [3,4]. The incidence of CD is increasing, partly due to improvements in detection and diagnostic testing [1]. CD results in the destruction of the small intestine and affects the nutritional status of a CD patient in many ways, including weight status, diet quality, and malabsorption, leading to nutrient deficiencies [5]. Currently, the only effective treatment is lifelong adherence to a gluten-free diet (GFD), which excludes foods that contain the gluten molecules found in wheat, barley, and rye [6,7]. Patients must be aware of gluten-containing foods and must be able to read and understand food labels to choose suitable gluten-free foods [8,9,10]. Strict adherence to GFD is crucial for minimizing negative health outcomes.

In Saudi Arabia, national efforts have been made to improve the nutritional care of CD patients through the Ministry of Health GFD program and the Saudi Arabia Celiac Association, which supports CD patients by providing GF food products as well as financial support to help with the cost of purchasing GF foods [11]. Additionally, the policies set by the Saudi Food and Drug Authority concerning nutrition facts require marking GF foods with a “gluten-free” label. There are limited data concerning GFD adherence among adult CD patients in Saudi Arabia. Thus, we aimed in this pilot study to assess the knowledge level relating to GFD. Additionally, the levels of adherence to GFD and barriers to adherence to GFD were explored.

## 2. Materials and Methods

### 2.1. Study Population

A total of 1170 patients were tested for CD in King Fahad Hospital, Madinah, Saudi Arabia, between 2021 and 2022. The exclusion criteria included negative immunoglobulin A (IgA)/immunoglobulin G (IgG) in both tissue transglutaminase and anti-gliadin antibody tests, age under 19 years, and a recent diagnosis (less than two months prior to data collection). The minimum sample needed for this study was 36 patients based on an expected proportion of CD of 2.5% [12] and a 5% total width of the confidence interval [13].

### 2.2. Data Collection

This cross-sectional study invited 112 CD patients to participate, of whom 36 agreed to participate. After agreeing to be part of this study, CD patients were asked to complete an online survey to collect data concerning sociodemographic characteristics (age, gender, marital status, family income, education level, work status, place of residency), height (in cm) and weight (in kg) to calculate body mass index (BMI), awareness of the support program offered to CD patients by the government of Saudi Arabia, knowledge related to GFD, adherence to the GFD, and barriers to adherence.

### 2.3. Validation of Tools Used

The translation was conducted by two bilingual experts in the nutrition field, proficient in both English and Arabic, who performed forward and backward translations to retain the original meaning of all items. Language validity was ensured by adapting the wording to align with cultural and linguistic norms familiar to Arabic speakers. Additionally, content validity was assessed by the same two experts in the field, who evaluated each item for relevance and appropriateness. To further enhance clarity, a professional outside the field validated the clarity and meaning of the items to ensure they were easily understood by the target population. Modifications were made to the methods section to improve the clarity of the process used to translate items.

### 2.4. Assessment of Knowledge Related to Support Program and Gluten-Free Diet

Data concerning knowledge related to the support program offered to CD patients by the government of Saudi Arabia and knowledge related to GFD were collected using the online questionnaire. The items used in the online questionnaire were 13 items that were designed to assess participants’ awareness of GF programs provided by the government, familiarity with the concept of a gluten-free diet, and membership in the Saudi Celiac Society. It also evaluated participants’ understanding of gluten-containing foods and the importance of dietary practices.

### 2.5. Assessment of Adherence to a Gluten-Free Diet

Data concerning adherence to GFD were collected using a modified version of the GFD adherence tool developed by Leffler and colleagues [14]. The tool was translated into Arabic and reviewed by two nutrition experts for content validation. This section includes 7 items with a total score of 35 points. The items evaluate physical symptoms, including low energy and headaches, behavioral and cognitive aspects of adherence, and attitudes toward accidental gluten exposure. One item specifically addresses intentional gluten consumption over the past four weeks. Responses are scored on a 5-point Likert scale, with higher scores indicating better adherence. 

### 2.6. Assessment of Barriers to Adhering to a Gluten-Free Diet

Data concerning barriers to adhering to GFD were collected using a modified version of the barriers affecting adherence to GFD tool developed by Muhammad and his colleagues [15]. The tool was translated into Arabic and validated by two experts in the field of nutrition. This section includes 7 items with a total score of 35 points. Items assessed common challenges, including understanding food labeling, time constraints, taste dissatisfaction, and the high cost of GF foods. Additional items assess the role of healthcare professionals in prescribing and providing adequate GF products. Responses are rated on a 5-point Likert scale, with higher scores indicating greater perceived barriers to adherence to GFD. 

### 2.7. Statistical Analysis

Data were analyzed using SPSS (version 29). Categorical variables were described in terms of frequency and percentage (%), while continuous variables were described as mean ± standard deviation (SD). The Shapiro–Wilk test was used to assess the normality of distributions, and variables were normally distributed (score for adherence to GFD, *p* = 0.318; score for barriers to adhering to GFD, *p* = 0.771). An independent samples *t*-test and one-way ANOVA were used to compare the means of the scores for adherence to a GFD and barriers to adhering to a GFD across groups. All tests were two-tailed, with a confidence level of 95%.

## 3. Results

### 3.1. Characteristics of the Sample of Celiac Disease Patients

A total of 36 CD patients were included in the final analysis in this study. Detailed data concerning the characteristics of the sample of CD patients are presented in Table 1. Most of the CD patients were women (75.0%, n = 27), and 61.2% were aged between 19 and 40 years (n = 22). Two-thirds of the CD patients reported being married (63.9%, n = 23), and 44.4% (n = 16) reported a monthly income of <SAR 6000 (<USD 1600). A total of 58% of CD patients (n = 21) had a university degree or higher, while 55.5% (n = 20) were unemployed. Most of the study participants (88.9%, n = 32) were residing in Madinah (Table 1).

### 3.2. Factors Related to the Health and Lifestyle of Celiac Disease Patients

Detailed data concerning factors related to the health and lifestyle of the CD patients are presented in Table 2. More than half of the CD patients reported sleeping at least 7 h per night (58.3%, n = 21). A total of 33% of the CD patients were overweight or obese (n = 12), and 41.7% (n = 15) of them reported seeing a dietitian. The majority of the CD patients reported using nutritional supplements (66.7%, n = 24). More than three-quarters of the CD patients reported having other chronic diseases (83.3%, n = 30), while 41.7% (n = 15) reported having other autoimmune diseases, with thyroid dysfunction being the most frequently reported autoimmune disease (30.6%, n = 11).

### 3.3. Knowledge Related to a Gluten-Free Diet

Only one-third of the CD patients were aware of the Ministry of Health GFD program implemented in Saudi Arabia (33.3%, n = 12). Thirty-one percent of the CD patients reported receiving GF products from the Ministry of Health GFD program (n = 11), while 27.8% of them received financial support (n = 10). Thirty-one percent of the CD patients had been receiving these services for at least 1 month when the survey was administered (n = 11). Most of the CD patients who benefited from the Ministry of Health GFD program reported learning about it through a doctor or dietitian (n = 10). Three-quarters of the CD patients were aware of what GFD entails (77.8%, n = 28), and 91.7% (n = 33) correctly defined GFD. The majority of the CD patients understood the importance (very important) of adhering to GFD (80.0%, n = 29). In addition, 88.9% (n = 32) of the CD patients understood the importance (very important) of reading nutrition facts, while half of them (58.3%, n = 21) understood the importance (very important) of using special utensils for GF food. The percentages of CD patients who correctly identified the sources of GF foods were as follows: bran, 16.7% (n = 6); oats, 33.3% (n = 12); and rice, 63.9% (n = 23). The percentages of those who correctly identified the sources of gluten were as follows: wheat, 97.2% (n = 35); bread, 94.4% (n = 34); and barley, 88.9% (n = 32). Most of the CD patients agreed (strongly agreed/somewhat agreed) that eating foods like pasta affects CD (83.3%, n = 30). Only 10.2% (n = 12) of the CD patients reported being members of the Ministry of Health GFD program (Supplementary Table S1).

### 3.4. Associations with Adherence to and Barriers to Adherence to a Gluten-Free Diet

Descriptive data on CD patients’ adherence to GFD and the barriers to adherence are presented in Supplementary Tables S2 and S3. Analysis of health and lifestyle factors in relation to GFD adherence and barriers revealed that the mean adherence scores were comparable across the different groups. Similarly, the mean scores for barriers to adherence to GFD did not significantly differ between groups (Table 3).

### 3.5. Knowledge Related to the Support Program and a Gluten-Free Diet in Relation to Adherence to and Barriers to Adherence to Gluten-Free Diet

**Figure 1:**
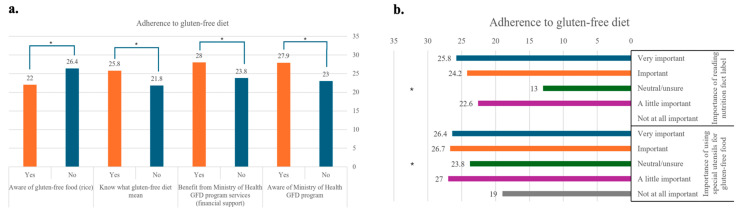
Knowledge related to adherence to a gluten-free diet. (**a**). Responses to survey questions assessing participants’ knowledge (n = 36) regarding key aspects of adherence to a gluten-free diet. Questions included awareness of the Ministry of Health GFD program, perceived benefits from its services (financial support), understanding of what a gluten-free diet means, and knowledge of gluten-free foods (oats). Response options were “Yes” and “No”. Data are presented using colored bars (orange: yes, blue: no). Significant differences were observed in all items: awareness of the Ministry of Health GFD program (*p*-value = 0.006), benefits from program services (financial support) (*p*-value = 0.014), knowledge of the meaning of gluten-free diet (*p*-value = 0.032), and identification of gluten-free foods (oats) (*p*-value = 0.005). (**b**). Survey responses (n = 36) evaluating the perceived importance of specific practices related to gluten-free diet adherence. Participants rated the importance of reading nutrition fact labels and using separate utensils for gluten-free food preparation. Response options ranged from blue (very important), orange (important), green (neutral/unsure), purple (a little important), and grey (not at all important). Statistically significant findings were observed for both practices: importance of label reading (*p*-value = 0.034) and use of special utensils (*p*-value = 0.015). * statistically significant.

The mean score of adherence was higher among CD patients who were aware of the Ministry of Health GFD program compared to those who were not aware of the program. CD patients who received financial support from the Ministry of Health GFD program had a higher mean score of adherence than those who did not receive financial support. In addition, the mean score of adherence to GFD was higher among CD patients who knew the definition of GFD than among patients who did not know the definition. CD patients who understood the importance of reading nutritional information on food labels reported a higher mean adherence score to GFD compared to those who did not. The mean score of adherence to GFD was lower among CD patients who distinguished GF foods (e.g., oats) than among patients who did not. CD patients who understood the importance of using special utensils for GF foods had a higher mean score of adherence to GFD than those who did not understand this (Supplementary Table S4 and Figure 1).

**Figure 2:**
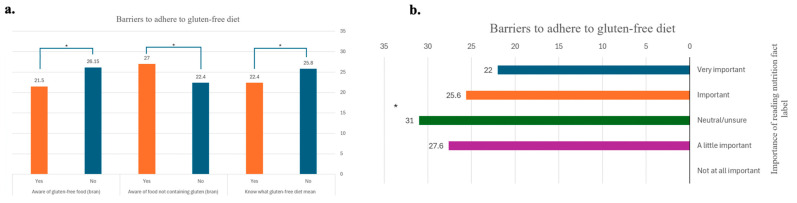
Knowledge related to barriers to adherence to a gluten-free diet. (**a**). Survey responses (n = 36) assessing participants’ knowledge related to gluten-free dietary concepts and their association with reported barriers to adherence. This figure includes three yes/no items addressing understanding of the gluten-free diet and identifying whether bran and rice contain gluten. Responses were categorized as “Yes” or “No” and are displayed using colored bars (orange = yes, blue = no). Statistically significant differences were found across all items: understanding GFD (*p*-value = 0.028), bran knowledge (*p*-value = 0.008), and rice knowledge (*p*-value = 0.001). (**b**). Survey responses (n = 36) assessing the perceived importance of reading nutrition fact labels and their association with barriers to following a gluten-free diet. Participants answered using a 5-point Likert scale: blue (very important), orange (important), green (neutral/unsure), purple (a little important), and grey (not at all important). A statistically significant difference in barrier scores was observed across response groups (*p*-value = 0.003). * statistically significant.

The mean score of barriers to adhering to GFD was higher among CD patients who did not know the definition of GFD than among patients who knew the definition and was also higher among CD patients who were unsure about the importance of reading nutritional facts on food labels than among other patients. A higher mean score of barriers to adhering to GFD was reported among CD patients who did not distinguish foods containing gluten (e.g., bran) from patients who did. Furthermore, the mean score of barriers to adhering to GFD was higher among CD patients who did not distinguish GF foods (e.g., rice) than among patients who did (Supplementary Table S4 and Figure 2).

## 4. Discussion

The results of this study validate the significant role that knowledge, practicality, and organizational support play in gluten-free diet adherence. Most CD patients showed a significant knowledge of GFD and its significance, together with a great understanding of its definition and adherence. However, while most CD patients accurately identified common gluten sources and understood the impact of gluten-containing foods on their condition, their knowledge of gluten-free food sources was inadequate. Patients who distinguished specific gluten-free foods like oats reported lower adherence scores, which may reflect confusion or misinformation about certain borderline products. Furthermore, individuals who acknowledged the significance of utilizing separate utensils for gluten-free food showed greater adherence, indicating that practical understanding may improve patients’ compliance. Similarly, recognizing the importance of reading nutritional labels was positively correlated with a significant adherence, highlighting the importance and impact of patients’ complete understanding of GF products on their compliance. In contrast, patients with insufficient understanding and those who were uncertain about the importance of reading food labels exhibited high barrier ratings. Thus, a complete understanding of GFD along with all related aspects, such as gluten-free products and gluten-containing products, is crucial to facilitate adherence to GFD. Therefore, agreeing with other findings noted in celiac patients worldwide [10,16,17], robust adherence correlates with patients’ comprehension of the diet and availability of educational resources and support networks.

Individuals with celiac disease face several significant barriers that hinder their adherence to the GFD. While most patients’ responses indicated awareness and understanding of the importance of adherence, a major barrier was the practicality of adhering to the GFD. 58.3% reported difficulty in distinguishing gluten-containing products from gluten-free products, suggesting a need for dietary education and support. Additionally, 30.5% of CD patients considered food labeling confusing, which could result in anxiety around food choices and possible gluten exposure. Another factor was the time requirement for selecting and preparing gluten-free meals; thus, time constraints were considered a barrier for working individuals and those with other responsibilities. Furthermore, 72.2% of the patients revealed the unpleasant taste of gluten-free products as a barrier to long-term adherence and 94% of the patients reported the high cost of the GFD as another barrier to adherence and considered it a substantial burden. These results suggest that in addition to medical guidance, celiac patients would benefit from practical support such as budget-friendly meal planning, improved food labeling, and increased availability of pleasant and affordable gluten-free options. Many studies show that practical issues, especially those about cost, time, taste, and recognizing gluten-free foods, make it hard for celiac patients to stick to their diet and need specific solutions to help them follow their diet long-term [16,17,18]. A study completed on local stores and supermarkets in Jeddah province, Saudi Arabia, looked at the selection and prices of gluten-free foods and found that gluten-free products are more expensive and harder to find compared to foods that contain gluten. This shows how important the food industry is in making gluten-free products more available and affordable to help with financial difficulties [19]. This highlights the important role the food industries play in the availability and affordability of GF products to ease financial challenges.

While 72% of the CD patients included in this study recognized the importance of following GFD, limited adherence was observed among our study sample. Several factors can contribute to adherence to GFD, including knowledge of support programs, food sources that contain gluten, cooking skills, and how to read nutrition facts on food labels [9]. Reading nutrition fact labels is an important practice that needs to be followed during grocery shopping, but the Saudis do not commonly follow this practice. A survey conducted among university students in Riyadh, Saudi Arabia, showed that although 98% of respondents confirmed the importance of food labels, only 20% of them used them daily [20]. Increasing awareness among CD patients in Saudi Arabia should be promoted to improve adherence to GFD. Additionally, long-term adherence to GFD often might result in nutritional deficiencies, most commonly in vitamin D, calcium, iron, folic acid, and vitamin B12 [5,21,22]. Our results indicated that two-thirds of CD patients used supplements, which indicates a high awareness of the importance of supplement use among CD patients.

The Ministry of Health GFD program was initiated to support CD patients in Saudi Arabia by providing them with free GF products and financial support [11]. However, one of the main findings in this study was the insufficient knowledge about the Ministry of Health GFD program observed among CD patients, with only a small proportion of CD patients reporting benefiting from the program. Patients informed about the Ministry of Health GFD initiative and who obtained financial aid exhibited markedly greater adherence levels, underscoring the importance of organizations and financial support in facilitating CD. The limited benefit of this program might be due to the limited knowledge of its existence, as the GFD program was founded in 2018. CD patients who were diagnosed before then are less likely to be aware of the program, especially if they do not see dietitians or family physicians. The role of dietitians, who typically discuss food-related challenges with patients, is essential to improve knowledge related to GFD and increase adherence to the prescribed diet. This study indicated the limited involvement of dietitians and the healthcare team in monitoring CD patients. While this study shows no link observed between visiting a dietitian and both adhering to GFD and barriers to adhering to GFD, other studies have found that the dietary education provided by dietitians improved adherence to GFD and improved dietary balance [23,24,25]. The lack of association reported in our study might be explained by the limited number of CD patients involved in this study. Healthcare practitioners, particularly dietitians, play an essential role in promoting awareness among celiac patients about the Ministry of Health GFD program, while also providing personalized dietary plans, ongoing guidance to support adherence, and improving overall health outcomes [25]. Therefore, patients diagnosed with CD should be referred to dietitians to improve their nutritional status. Another key strategy to enhance patient awareness of the program is through the Saudi Arabia Celiac Association. The association’s mission is to support celiac patients in adapting to their new lifestyle and increasing awareness of the condition both within the broader community and among patients. Moreover, by collaborating with key stakeholders, the association can frame policies and implement initiatives that provide continuous support for celiac patients.

This study contributes to exploring the knowledge, adherence, and barriers related to the GFD among adult celiac disease patients in Saudi Arabia, specifically in the Madinah region. One of the strengths of this study is its comprehensive approach, assessing multiple measurements of GFD management, exploring associations between adherence and factors related to comprehensive knowledge and practicality, and national support program awareness. By evaluating awareness of the Ministry of Health’s GFD program, this study provides insight into how awareness about governmental support may improve dietary behavior, influencing practical implications for policy and healthcare interventions. This study has some limitations, including the use of a small number of patients. Using data from a single center also limits the generalizability of this study’s findings. Additionally, the cross-sectional nature of this study limits its ability to determine the relation or observe changes over time [26]. However, it is important to note that this was a pilot study, and the primary aim was to evaluate the feasibility and appropriateness of the assessment tool in a specific population [27]. Thus, using a small sample is considered acceptable and provides valuable insights for larger-scale future studies.

## 5. Conclusions

Our findings suggest the need for comprehensive public health strategies that extend beyond education and include financial aid, better food labeling, and broader advertising of government support programs. To improve the quality of life and long-term health outcomes for people with celiac disease, policymakers and healthcare providers should work together to lower practical barriers to GFD adherence and increase the effectiveness of current initiatives. Healthcare practitioners, along with national associations such as the Saudi Celiac Association, should provide initiatives and programs that contribute to increasing CD patients’ awareness about the Ministry of Health’s GFD program, provide reliable sources for information, and collaborate with food industries to ease the barriers to adhering to GFD in celiac patients. Future research should focus on gathering data at the national level and generating longitudinal data to investigate the long-term impacts of factors associated with GFD.

## Figures and Tables

**Table 1:** Sample characteristics (n = 36).

Variable	n	%
Gender		
Male	9	25.0
Female	27	75.0
Age group		
19–30 years	11	30.6
31–40 years	11	30.6
41–50 years	9	25.0
>50 years	5	13.9
Marital status		
Single	13	36.1
Married	23	63.9
Family monthly income		
<SAR 6000	16	44.4
SAR 6000–10,999	10	27.8
SAR 11,000–15,999	2	5.56
≥SAR 16,000	8	22.2
Education level		
<High-school	4	11.1
High-school/Diploma	11	30.6
Undergraduate degree	18	50.0
Postgraduate degree	3	8.33
Work status		
Student	4	11.1
Employed	12	33.3
Unemployed/Retired/Other	20	55.5
Place of residency		
Madinah	32	88.9
Other cities in Madinah province	4	11.1

SAR: Saudi Riyal. USD 1 = SR 3.75.

**Table 2:** Factors related to the health and lifestyle of celiac patients (n = 36).

Variable	n	%
Sleeping hours per day		
<7 h	15	41.7
≥7 h	21	58.3
Weight status		
Underweight (<18.5 kg/m^2^)	7	19.4
Healthy weight (18.5–24.9 kg/m^2^)	17	47.2
Overweight (25–29.9 kg/m^2^)	6	16.7
Obesity (≥30 kg/m^2^)	6	16.7
Visited dietitian		
No	21	58.3
Yes	15	41.7
Frequency of visiting dietitian		
Never	21	58.3
Once	8	22.2
More than once	7	19.4
Supplement use		
No	12	33.3
Yes	24	66.7
Other chronic diseases		
No	6	16.7
Yes	30	83.3
Diabetes, yes	8	26.6
Hypertension, yes	3	10.0
Renal disease, yes	3	10.0
Other autoimmune diseases		
No	21	58.3
Yes	15	41.7
Thyroid diseases, yes	11	30.6
Arthritis, yes	4	11.1
Crohn’s disease, yes	1	2.78

**Table 3:** Factors related to the health and lifestyle of the celiac patients in relation to adherence and barriers to adhering to a gluten-free diet (n = 36).

Variable	Adherence to Gluten-Free Diet Score Out of 35	Barriers to Adhere to Gluten-Free Diet Score Out of 35
Sleeping hours per day		
<7 h	25.6 ± 4.03	21.7 ± 4.13
≥7 h	24.5 ± 5.17	24.2 ± 3.49
*p*-value	0.506	0.053
Weight status		
Underweight (BMI < 18.5 kg/m^2^)	23.2 ± 4.15	22.4 ± 4.99
Healthy weight (BMI 18.5–24.9 kg/m^2^)	26.8 ± 3.79	23.5 ± 3.34
Overweight (BMI 25–29.9 kg/m^2^)	22.6 ± 5.64	23.0 ± 4.42
Obese (BMI ≥ 30 kg/m^2^)	24.5 ± 6.09	23.1 ± 4.70
*p*-value	0.194	0.952
Visited dietitian		
No	24.1 ± 5.05	24.0 ± 3.49
Yes	26.2 ± 3.98	22.1 ± 4.35
*p*-value	0.189	0.163
Frequency of visiting dietitian		
Never	24.1 ± 5.05	24.0 ± 3.49
Once	25.3 ± 3.62	21.3 ± 3.37
More than once	27.1 ± 4.45	23.0 ± 5.41
*p*-value	0.328	0.278
Supplement use		
No	23.7 ± 4.55	24.3 ± 4.29
Yes	25.5 ± 4.74	22.6 ± 3.70
*p*-value	0.276	0.235
Other chronic diseases		
No	26.0 ± 4.73	21.1 ± 4.26
Yes	24.7 ± 4.74	23.6 ± 3.80
*p*-value	0.565	0.163
Diabetes		
No	25.0 ± 4.81	22.5 ± 3.69
Yes	24.8 ± 4.58	25.6 ± 3.99
*p*-value	0.948	0.048
Hypertension		
No	24.6 ± 4.78	23.3 ± 3.95
Yes	28.3 ± 1.52	21.6 ± 4.04
*p*-value	0.200	0.482
Renal disease		
No	25.2 ± 4.64	23.2 ± 4.05
Yes	22.3 ± 5.50	23.0 ± 2.64
*p*-value	0.317	0.920
Other autoimmune diseases (thyroid diseases, arthritis, Crohn’s disease)		
No	24.9 ± 5.50	24.1 ± 3.30
Yes	25.0 ± 3.45	21.9 ± 4.46
*p*-value	0.921	0.097
Thyroid diseases		
No	25.0 ± 5.37	23.8 ± 3.72
Yes	24.8 ± 2.82	21.7 ± 4.14
*p*-value	0.898	0.132
Arthritis		
No	24.6 ± 4.81	23.5 ± 3.81
Yes	27.5 ± 3.00	20.2 ± 4.03
*p*-value	0.260	0.110
Crohn’s disease		
No	25.1 ± 4.65	23.0 ± 3.85
Yes	19.0 ± 0.00	29.0 ± 0.00
*p*-value	0.202	0.138

Data presented in the table are mean ± SD. Independent samples *t*-test and one-way ANOVA test were performed to assess the significance level at alpha = 0.05.

## Data Availability

The data presented in this study are available on request from the corresponding author due to (Patient privacy).

## Supplementary Materials

The following supporting information can be downloaded at: https://www.mdpi.com/article/10.3390/healthcare13101208/s1, Table S1. Knowledge related to support programs and gluten-free diets. Table S2: adherence to a gluten-free diet; Table S3: barriers to adhere to gluten-free diet. Table S4. Knowledge related to gluten-free diet patients in relation to adherence and barriers to adhere to gluten-free diet.

## Abbreviations

The following abbreviations are used in this manuscript:

CD	Celiac disease
GFD	Gluten-free diet
GF	Gluten-free
TTG-IgA	Tissue Transglutaminase IgA
