# Supplementary material for: Knowledge, Adherence, and Barriers to Gluten-Free Diet Among Adults with Celiac Disease in Saudi Arabia: A Pilot Study at a Tertiary Hospital in Madinah, Saudi Arabia
Source: Healthcare (Basel). 2025 May 21;13(10):1208. doi: 10.3390/healthcare13101208 (PMC12111401; doi:10.3390/healthcare13101208)
Supplement: Supplementary file 1 [file healthcare-13-01208-s001.zip › healthcare-3613326-supplementary.pdf]

Supplementary Materials

Table S1. Knowledge related to support programs and gluten-free diets (n= 36).

Variable	n	%
Aware of Ministry of Health gluten-free diet program		
No	24	66.7
Yes	12	33.3
Benefit from Ministry of Health gluten-free diet program services (products)		
No	25	69.4
Yes	11	30.6
Benefit from Ministry of Health gluten-free diet program services (financial support)		
No	26	72.2
Yes	10	27.8
Start getting these services (products or financial support)		
More than a week ago	0	0.00
More than a month ago	2	5.56
More than 6 months ago	3	8.33
More than a year	6	16.7
Not applicable	25	69.4
Know about Ministry of Health gluten-free diet program from		
Doctor	7	19.4
Dietitian	3	8.33
Family/friends	2	5.56
Social media	4	11.1
Not applicable	20	55.6
Know what gluten-free diet mean		
No	8	22.2
Yes	28	77.8
Definition of gluten-free diet		
Diet based on excluding foods containing gluten which is substance found in wheat and barley	33	91.7
Diet rich in vegetables fruit low fat dairy product with whole grains fish poultry and nuts	3	8.33
Importance of following gluten-free diet		
Very important	26	72.2
Important	3	8.33
Neutral/unsure	4	11.1
A little important	3	8.33
Not at all important	0	0.00
Importance of reading food labels		
Very important	27	75.0
Important	5	13.9
Neutral/unsure	1	2.78
A little important	3	8.33
Not at all important	0	0.00
Foods not containing gluten (wheat, no)	35	97.2
Foods not containing gluten (bread, no)	34	94.4
Foods not containing gluten (barley, no)	32	88.9
Foods not containing gluten (bran, yes)	6	16.7
Foods not containing gluten (oats, yes)	12	33.3
Foods not containing gluten (rice, yes)	23	63.9
Importance of using special utensils for gluten-free food		
Very important	17	47.2
Important	4	11.1
Neutral/unsure	7	19.4

A little important	3	8.33
Not at all important	5	13.9
Effect of eating foods like pasta on celiac		
Strongly agree	23	63.9
Somewhat agree	7	19.4
Neither agree nor disagree	2	5.56
Somewhat disagree	4	11.1
Strongly disagree	0	0.00
Member of the Saudi Celiac Society		
No	24	66.7
Yes	12	10.2

Table S2. Adherence to gluten-free diet (n= 36).

#		5 points	4 points	3 points	2 points	1 point
		**None of the time**	**A little of the time**	**Some of the time**	**Most of the time**	**All the time**
**1**	Have you been bothered by low energy level during the past 4 weeks?	6 (16.7)	8 (22.2)	10 (27.8)	7 (19.4)	5 (13.8)
**2**	Have you been bothered by headaches during the past 4 weeks?	5 (13.8)	14 (38.8)	10 (27.8)	2 (5.56)	5 (13.8)
		**Strongly agree**	**Somewhat agree**	**Neither agree nor disagree**	**Somewhat disagree**	**Strongly disagree**
**3**	I am able to follow a gluten-free diet when dining outside my home	9 (25.0)	10 (27.8)	4 (11.1)	7 (19.4)	6 (16.7)
**4**	Before I do something I carefully consider the consequences	19 (52.8)	12 (33.3)	1 (2.78)	4 (11.1)	0 (0.00)
**5**	I do not consider myself a failure	12 (33.3)	17 (47.2)	3 (8.33)	1 (2.78)	3 (8.33)
		**Very important**	**Important**	**Neutral/ Unsure**	**A little important**	**Not at all important**
**6**	How important to your health are accidental gluten exposures?	12 (33.3)	9 (25.0)	12 (33.3)	1 (2.78)	2 (5.56)
		**0 (never)**	**1-2 times**	**3-5 times**	**6-10 times**	**>10 times**
**7**	Over the past 4 weeks, how many times have you eaten foods containing gluten on purpose?	14 (38.8)	11 (30.5)	4 (11.1)	6 (16.6)	1 (2.78)

Data presented in table are frequency (percentage).

Table S3. Barriers to adhere to gluten-free diet (n= 36).

#		5 points	4 points	3 points	2 points	1 point
		**Strongly agree**	**Somewhat agree**	**Neither agree nor disagree**	**Somewhat disagree**	**Strongly disagree**
**1**	I don't understand what foods I can and cannot eat	10 (27.8)	11 (30.6)	5 (13.9)	3 (8.33)	7 (19.4)
**2**	I don't understand the labeling of foods	6 (16.7)	5 (13.9)	6 (16.7)	6 (16.7)	13 (36.1)
**3**	I don't have the time to prepare different meals	9 (25.0)	18 (50.0)	2 (5.56)	4 (11.1)	3 (8.33)
**4**	Gluten-free foods have an unpleasant taste	7 (19.4)	19 (52.7)	4 (11.1)	1 (2.78)	5 (13.9)
**5**	Gluten-free foods are expensive to buy	25 (69.4)	9 (25.0)	2 (5.56)	0 (0.00)	0 (0.00)
		**Strongly disagree**	**Somewhat disagree**	**Neither agree nor disagree**	**Somewhat agree**	**Strongly agree**
**6**	Dietitian/General physician prescribes gluten-free foods	4 (11.1)	0 (0.00)	5 (13.9)	13 (36.1)	14 (38.9)
**7**	Dietitian/General physician prescribes sufficient amounts of gluten-free products	9 (25.0)	4 (11.1)	12 (33.3)	7 (19.4)	4 (11.1)

Data presented in table are frequency (percentage).

Table S4. Knowledge related to gluten-free diet patients in relation to adherence and barriers to adhere to gluten-free diet (n= 36).

Variable	Adherence to gluten-free diet Score out of 35	Barriers to adhere to gluten-free diet Score out of 35
**Aware of Ministry of Health GFD program**		
No	23.0 ± 4.39	23.8 ± 3.57
Yes	27.9 ± 3.96	21.9 ± 4.42
p-value	0.006*	0.162
**Benefit from Ministry of Health GFD program services (products)**		
No	23.9 ± 4.75	23.8 ± 4.07
Yes	27.2 ± 3.82	21.9 ± 3.39
p-value	0.050	0.187
**Benefit from Ministry of Health GFD program services (financial support)**		
No	23.8 ± 4.72	23.6 ± 4.10
Yes	28.0 ± 3.12	22.2 ± 3.42
p-value	0.014*	0.340
**Start getting these services (products or financial support)**		
More than a week ago	0.00 ± 0.00	0.00 ± 0.00
More than a month	30.0 ±1.41	22.0 ± 4.24
More than 6 months	25.6 ± 4.93	21.0 ± 2.64
More than a year	27.1 ± 3.81	22.3 ± 3.98
Not applicable	23.9 ± 4.75	23.8 ± 4.07
p-value	0.186	0.591
**Know about Ministry of Health gluten-free diet program from**		
Doctor	28.0 ± 3.65	23.0 ± 1.82
Dietitian	24.6 ± 5.03	21.3 ± 5.85
Family/friends	20.5 ± 10.6	24.0 ± 9.89
Social media	26.5 ± 2.38	20.5 ± 3.69
Not applicable	24.1 ± 4.49	24.0 ± 3.67
p-value	0.212	0.482
**Know what gluten-free diet mean**		
No	21.8 ± 5.19	25.8 ± 3.56
Yes	25.8 ± 4.24	22.4 ± 3.74
p-value	0.032*	0.028*
**Definition of gluten-free diet**		
Diet based on excluding foods containing gluten which is substance found in wheat and barley.	24.6 ± 4.65	22.8 ± 3.85
Diet rich in vegetables fruit low fat dairy product with whole grains fish poultry and nuts.	29.0 ± 3.60	27.3 ± 2.30
p-value	0.123	0.057
**Importance of following gluten-free diet**		
Very important	25.6 ± 4.38	22.5 ± 3.65
Important	20.6 ± 4.16	24.6 ± 1.15
Neutral/unsure	25.5 ± 1.73	23.2 ± 5.90
A little important	22.6 ± 9.07	27.6 ± 3.21

Not at all important	0.00 ± 0.00	0.00 ± 0.00
p-value	0.287	0.169
**Importance of reading nutrition fact label**		
Very important	25.8 ± 3.15	22.0 ± 3.47
Important	24.2 ± 6.49	25.6 ± 2.07
Neutral/unsure	13.0 ± 0.00	31.0 ± 0.00
A little important	22.6 ± 9.07	27.6 ± 3.21
Not at all important	0.00 ± 0.00	0.00 ± 0.00
p-value	0.034*	0.003*
**Foods not containing gluten (wheat, no)**		
No	24.7 ± 4.50	23.1 ± 3.97
Yes	34.0 ± 0.00	25.0 ± 0.00
p-value	0.050	0.653
**Foods not containing gluten (bread, no)**		
No	24.6 ± 4.56	23.2 ± 4.02
Yes	30.0 ± 5.65	23.5 ± 2.12
p-value	0.121	0.920
**Foods not containing gluten (barley, no)**		
No	24.5 ± 4.64	23.0 ± 3.94
Yes	28.0 ± 4.54	24.5 ± 4.12
p-value	0.175	0.498
**Foods not containing gluten (bran, yes)**		
No	25.0 ± 4.09	22.4 ± 3.74
Yes	24.5 ± 7.55	27.0 ± 2.44
p-value	0.792	0.008*
**Foods not containing gluten (oats, yes)**		
No	26.4 ± 3.43	22.7 ± 3.96
Yes	22.0 ± 5.57	24.2 ± 3.81
p-value	0.005*	0.273
**Foods not containing gluten (rice, yes)**		
No	23.9 ± 6.47	26.15 ± 2.79
Yes	25.5 ± 3.35	21.5 ± 3.51
p-value	0.321	<0.001*
**Importance of using special utensils for gluten-free food**		
Very important	26.4 ± 2.93	22.4 ± 3.85
Important	26.7 ± 4.03	24.2 ± 1.70
Neutral/unsure	23.8 ± 5.55	23.4 ± 5.31
A little important	27.0 ± 6.55	24.3 ± 5.50
Not at all important	19.0 ± 4.12	24.0 ± 3.24
p-value	**0.015***	0.868
**Effect of eating foods like pasta on celiac**		
Strongly agree	26.4 ± 3.96	22.0 ± 4.06
Somewhat agree	22.0 ± 5.68	25.4 ± 2.69
Neither agree or not	24.5 ± 0.70	26.0 ± 1.41
Somewhat disagree	22.0 ± 5.35	25.0 ± 3.36
Strongly disagree	0.00 ± 0.00	0.00 ± 0.00
p-value	0.077	0.094
**Member of the Saudi Celiac Society**		
No	23.9 ± 4.35	23.0 ± 4.10

Yes	27.0 ± 4.83	23.5 ± 3.70
p-value	0.055	0.702

* Significance level at alpha= 0.05.
